# *Protospirura muricola* Gedoelst, 1916 (Spiruridae) in eastern spiny mice (*Acomys dimidiatus*): more than a decade of stability in frequency distributions of worm burdens, and evidence for both frequent low-dose and clumped transmission

**DOI:** 10.1017/S0031182026102492

**Published:** 2026-08

**Authors:** Jerzy M. Behnke, Joseph A. Jackson, Anna Bajer, Jolanta Behnke-Borowczyk, Eman M.E. Mohallal, Francis Gilbert

**Affiliations:** 1 School of Life Sciences, University of Nottingham, University Park, Nottingham, UK; 2 School of Science, Environment and Engineering, University of Salford, Salford, UK; 3 Department of Eco-Epidemiology of Parasitic Diseases, Institute of Developmental Biology and Biomedical Sciences, Faculty of Biology, University of Warsaw, Warsaw. Poland; 4 Department of Entomology and Forest Pathology, Faculty of Forestry and Wood Technology, Poznań University of Life Sciences, Poznań, Poland; 5 Department of Animal Physiology, Desert Research Centre, El Matareya, Cairo, Egypt

**Keywords:** *Acomys dimidiatus*, eastern spiny mice, frequency distribution of worm burdens, low-dose transmission, package transmission, *Protospirura muricola*, Spiruridae

## Abstract

We examined the frequency distributions of worm burdens and worm biomass of the dominant gastric nematode, *Protospirura muricola*, of eastern spiny mice (*Acomys dimidiatus*) surveyed at 4-year intervals over a period of 12 years in two montane wadis in St. Kathrine's Protectorate in S. Sinai, Egypt. Whilst the frequency distribution of worm burdens differed significantly between the mice from the 2 wadis, variation between years was not significant among mice from each wadi. The frequency distribution of *P. muricola* worm burdens did not differ significantly between the sexes, despite female mice carrying significantly higher worm burdens, but showed a marked difference between juvenile and mature mice. The biomass of *P. muricola* carried by individual mice varied considerably, with two mice from Wadi Gharaba carrying biomasses of over 1 gm, representing over 2.5% of total body weight. Biomass showed significant positive covariation with worm burdens, and heavy biomass and worm burdens were most likely among the older mature female mice. The frequency distribution of individual worm weights indicated that frequent low-dose transmission was common, with rarer instances of higher-dose, package transmission from insect vectors of this parasite.

## Graphical abstract

**Figure:**
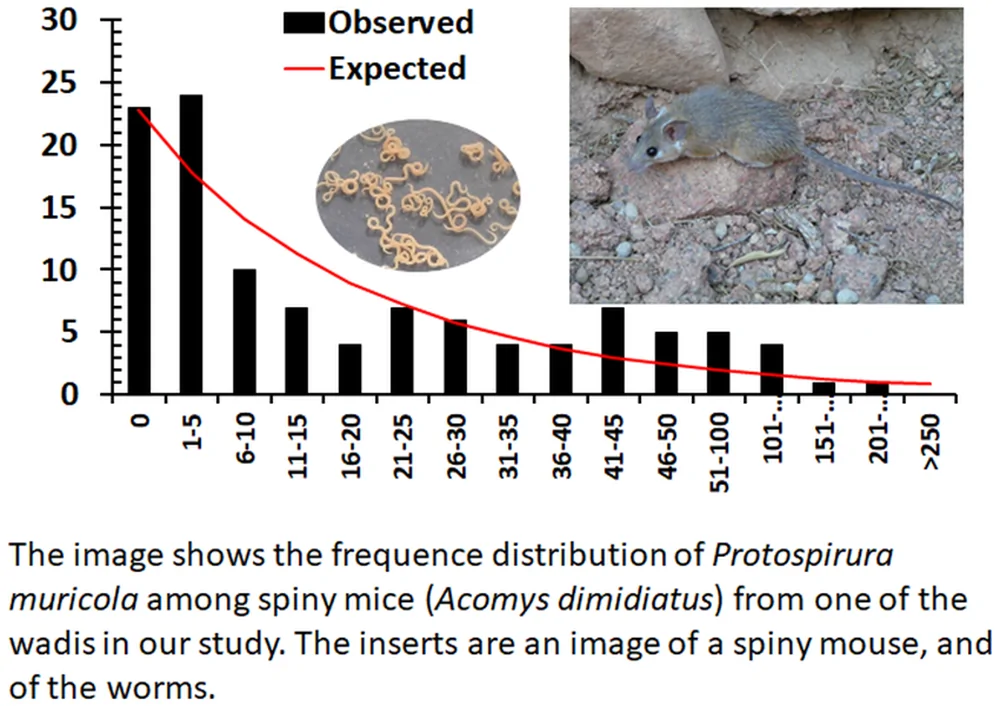

## Introduction

The over-dispersed or aggregated distribution of worm burdens among hosts in a population is generally regarded as one of the hallmarks of helminth infection (Crofton, 1971; Anderson and May, 1978; Shaw et al., 1998; Haukisalmi and Henttonen, 1999). Numerous studies have shown that worm burdens are not distributed randomly among hosts, but rather that large proportions of the worms in a population aggregate in relatively few hosts (Pennycuik, 1971; Haukisalmi, 1986; Shaw et al., 1998; Bajer et al., 2005). Among the first to recognize this characteristic feature in human helminth infections were Croll and Ghadirian (1981), who coined the term ‘wormy persons’ for the relatively small proportion of individuals who carried heavy worm burdens and an exceptionally high proportion of all the worms recovered from their study population. This overdispersion of worms in host populations is usually best accounted for by the negative binomial distribution (Crofton, 1971; Wilson and Grenfell, 1997), although other approaches are also possible, such as zero-inflated or hurdle models (e.g. Nketia and De Souza, 2024). Various studies have explored the interrelationships between measures of parasite infection (prevalence and abundance), sample size and parameters of aggregation in attempts to explain its biological basis (Anderson and Gordon, 1982; Gregory and Woolhouse, 1993).

In this paper, we look closely at the frequency distribution of worm burdens and worm biomasses in spiny mice (*Acomys dimidiatus*) infected naturally with the spirurid gastric nematode *Protospirura muricola.* This nematode is one of 14 known species in the genus *Protospirura*, 8 of which are parasites of murid rodents and is geographically the most widespread (see Miquel et al., 2023 for a detailed current review of the genus). *Protospirura muricola* has been recorded in tropical and subtropical regions of the world, including Africa (Baylis, 1928; Scharff et al., 1996; Asakawa and Nicolas, 2003), Asia (Tubangui, 1931), S. America (Campos and Vargas, 1978) and the Caribbean Islands (De León, 1964; Waugh et al., 2006). While often reported from rats (Gedoelst, 1916; Tubangui, 1931; De León, 1964; Campos and Vargas, 1978), *P. muricola* parasitizes a large range of rodents predominantly Muridae (Baylis, 1928; Smales et al., 2009) but also Sciuridae and Bathyergidae (Scharff et al., 1996; Smales et al., 2009) and has even been recorded in captive and wild primates, Lorisidae and Ateidae (Foster and Johnson, 1939; Smales et al., 2009; Petrášová et al., 2010; Navarro-serra et al., 2021). A comprehensive list of hosts is provided by Smales et al. (2009).

Transmission of *P. muricola* is via arthropod intermediate hosts in which the first three larval stages of the parasite develop (Quentin, 1969). The most commonly reported arthropod hosts are insects, mostly cockroaches of various species (Dictyoptera; Campos and Vargas, 1977), although it also develops to the infective third larval stage (L3) in earwigs (Dermaptera; Quentin, 1969) and tenebrionid beetles (Coleoptera; Lowrie et al., 2004). The mammal-infective L3 encysts in the haemocoel of the intermediate host (Quentin, 1969) and can survive in this state for over 600 days (Schutgens et al., 2015).

In earlier studies of the helminths of spiny mice (*A. dimidiatus*) in Egypt (Behnke et al., 2000, 2004, 2019), we encountered exceptionally heavy infections with *P. muricola* in some of the sites where we trapped the hosts. Here, we report a population survey of *P. muricola*, providing novel ecological information for this understudied species and confirming its tendency towards an aggregated host distribution. Building on our earlier studies in which we documented the stability of measures of infection with *P. muricola* over time, we focus on the mice from two sites where prevalence was particularly high (Wadis Gharaba and Tlah) and assess whether the degree of aggregation of *P. muricola* was consistent over successive surveys conducted at 4-year intervals over more than a decade in each of these wadis. We also examine the pattern of aggregation in the sexes and age classes, in order to identify subsets of the mouse population that were most at risk of infection. Our data additionally provide unusual insight into the origin of overdispersion in this system via aggregated transmission, with this being possible to infer from the detailed parasite weight-frequency distributions that were recorded in the study. Thus, examination of individual *P. muricola* worms in moderately and heavily infected mice showed that while some mice harboured a large range of worm sizes suggestive of frequent or trickle low-dose exposure to infective stages, others showed a bimodal or even multimodal distribution pattern when worms were assessed by weight. We detail these distribution patterns and assess the degree to which hosts are exposed to low doses of infective larvae, versus larger packages of larvae from heavily infected insects, reflecting clumped transmission.

## Materials and Methods

### Study sites and sources of data

We utilized the database of Behnke et al. (2019), which entails long-term spatiotemporal data on the helminth parasites of spiny mice from four montane wadis in St. Katherine's Protectorate in the South Sinai Peninsula of Egypt. Four surveys were conducted in late summer months at four-year intervals (in 2000, 2004, 2008 and 2012). Here, we concentrate on the dominant, most abundant nematode parasite, *P. muricola*, in the two wadis where prevalence was highest (Wadi Gharaba and Wadi Tlah). A full description of these wadis, together with GPS data and a map of the area are provided in Behnke et al. (2004), and aspects of local biodiversity can be found in Zalat and Gilbert (1998) and Zalat et al. (2001).

### Terminology and collection of spiny mice

We refer to *Acomys dimidiatus* (Cretzschmar, 1826) for eastern spiny mice from this region of Egypt following Cassola (2016a) and not *A. cahirinus* (Geoffroy, 1803) (Cairo spiny mouse; Cassola, 2016b) as in some earlier studies from the region (Myers, 1961; Ward and Nelson, 1967; Greenberg, 1969), since the latter species is now known to occur across N. Africa, rather than on the north-eastern side of the Suez Canal.

The methods used for trapping rodents and for sampling and processing trapped animals have all been fully described previously (Behnke et al., 2004). Age categories were established as described earlier using principal components analysis (PCA) of a range of morphological measures including body weight and dried eye lens weight (Behnke et al., 2004) and based on all the mice from all four wadis as utilized by Behnke et al. (2019). Two age classes were established, and these were used in statistical models for consistency with earlier published analyses. Age class 1 mice were immature juveniles, while age class 2 mice were all young and breeding adults.

However, to obtain a more nuanced scale corresponding to increasing host age (and therefore a continuous data proxy for host age) for analysis of covariance, we conducted PCAs, confined to the mice in the current study from Wadis Gharaba and Tlah. Separate PCAs were conducted on males and females (after exclusion of pregnant animals) based on host weight, femur length, body length and dried eye lens weight. For male mice, principal component 1 (PC1) accounted for 85.7% of the variance and showed high correlation with the fitted factors (*R* for weight = 0.974, body length = 0.952, femur length = 0.819 and dried eye lens weight = 0.950). For non-pregnant female mice, PC1 accounted for 83.6% of the variance and showed high correlation with the fitted factors (*R* for weight = 0.959, body length = 0.972, femur length = 0.829 and dried eye lens weight = 0.890). Note that sample sizes in these analyses were slightly smaller than in other statistical models because for 15 mice we lacked measurements of some of the components fitted to the PCAs.

### Treatment of worms

Worms from the surveys in 2000, 2004 and 2008 were stored in formalin. Those from the survey in 2012 were stored in 70% ethanol. Worms from the mice sampled in 2012 were weighed to the nearest 0.1 mg, after removal from holding tubes, and brief blotting on filter paper (Behnke et al., 2000).

### Statistical analysis

Prevalence values (percentage of animals infected, based on presence/absence of parasites and hence binomially distributed data) are given with 95% confidence limits (CL_95_, in square brackets in the text), calculated by bespoke software based on the tables of Rohlf and Sokal (1995). Abundance of infection (including both infected and non-infected animals) and intensity of infection (infected animals only) are summarized by arithmetic means as defined by Bush et al. (1997) and Margolis et al. (1982), and standard errors of the mean (S.E.M.) are provided.

The degree of aggregation in the data was calculated by the index of discrepancy (*D*) as described by Poulin (1993) and the index of dispersion (*I*, variance to mean ratio). Frequency distributions of raw values were tested for goodness of fit to Gaussian, negative binomial, positive binomial and Poisson models by *χ*^2^ as described by Elliott (1977), and the negative binomial exponent *k* is provided. The acceptability of parametric models was evaluated through the goodness of fit of residuals from minimum sufficient Generalized Linear Models to the distributions listed above, through Q-Q plots and through estimation of the total deviance accounted for.

The statistical approach adopted has been documented comprehensively in our earlier publications (Behnke et al., 2004, 2008; Bajer et al., 2005). For analysis of prevalence, we used maximum likelihood techniques based on log-linear analysis of contingency tables in the software package IBM SPSS Statistics Version 28 (IBM Corporation). This approach is based on categorical values of the factors of interest, which are used to fit hierarchical log-linear models to multidimensional cross-tabulations using an iterative proportional-fitting algorithm that detects associations among the factors, one of which may be presence/absence of infection. Initially, full factorial models were fitted, incorporating as factors SEX (two levels, males and females), AGE (two levels, immature and mature animals), YEAR of study (four levels, 2000, 2004, 2008 and 2012), and SITE (two levels, Wadis Gharaba and Tlah). The presence or absence of parasites (INFECTION) was considered as a binary factor. All these five factors were fitted initially and beginning with the most complex model involving all possible main effects and interactions, those combinations that did not contribute significantly to explaining variation (alpha = 0.05) were eliminated stepwise beginning with the highest-level interaction (backward selection procedure). A minimum sufficient model (MSM) was then obtained, for which the likelihood ratio of χ^2^ was not significant, indicating that the removal of all remaining parameters significantly reduced the fit of the model. The importance of each term in interactions involving INFECTION in the final model was assessed by the probability that its exclusion would alter the model significantly, and these values are given in the text, assessed by a likelihood ratio test between nested models with and without each factor of interest. The remaining terms in the final model that did not include INFECTION (for example, variation among sites in the number of animals of each sex sampled [SITE x SEX]) are not given but can be made available from the authors on request.

For analyses of quantitative data, we used a Generalized Linear Model with negative binomial error structure, implemented in R version 4.5.2 (R Core Development Team). The full factorial models that converged satisfactorily were simplified using the STEP procedure and tested for significance using deletion of terms beginning with the highest order interaction by comparing models with or without that interaction, then models based on main effects plus 2- and 3-way interaction by deletion of 3-way interactions in turn, and so on until each main effect was evaluated in a model that only comprised all main effects. An MSM was then fitted (all significant interactions and main effects, plus any main effects that featured in interactions), and the process was repeated to obtain values for changes in deviance, test statistics (likelihood ratio, *LR*) and probabilities.

Frequency distributions of mice in worm burden classes were compared between sites and for each site between years, between sexes and age classes by the Anderson-Darling *k*-sample test in the *kSamples* package of R. For covariance of data, we used Pearson’s or Spearman’s test in R depending on whether the data met the requisite assumptions.

## Results

### Summary statistics

Prevalence, abundance, intensity and range of worm burdens are given in Table 1, for mice from each of the two wadis and combined. Prevalence was significantly higher for mice from Wadi Gharaba (*χ*^2^_1_ = 8.171, *P* = 0.004). Abundance was also higher (*LR*_1,237_ = 35.190, *P* < 0.0001). Additionally, there was a highly significant difference in prevalence (*χ*^2^_1_ = 48.04, *P* < 0.001) and abundance (*LR*_1,237_ = 36.144, *P* < 0.0001) between the two age classes and between the sexes (for prevalence *χ*^2^_1_ = 5.29, *P* = 0.021; for abundance *LR*_1,237_ = 8.219, *P* = 0.004). Neither prevalence (*χ*^2^_3_ = 2.73, *P* = 0.44) nor abundance (*LR*_3,234_ = 2.046, *P* = 0.563) differed significantly between the years. There were no significant interactions between these factors in models for prevalence or abundance.

**Table 1.** Summary statistics for *Protospirura muricola* in Wadis Gharaba and Tlah, and combined Table 1 long description.

Data subset	No. of mice	Prevalence	Abundance	No. infected	Intensity	Range
Wadi Gharaba	112	79.5 (72.93–84.89)	23.8 ± 3.38	89	29.9 ± 4.00	0–227
Wadi Tlah	129	62.8 (54.93–70.06)	7.3 ± 1.35	81	11.6 ± 2.01	0–143
Year 2000	32	71.9 (57.50–83.40)	15.7 ± 4.39	23	21.9 ± 5.63	0–122
Year 2004	59	74.6 (64.33–82.79)	15.9 ± 4.34	44	21.4 ± 5.61	0–227
Year 2008	66	65.2 (53.89–75.20)	15.6 ± 3.23	43	24.0 ± 4.48	0–122
Year 2012	84	71.4 (58.82–81.68)	13.4 ± 2.92	60	18.8 ± 3.89	0–176
Age, Class 1	86	43.0 (30.89–55.94)	5.2 ± 1.27	37	12.1 ± 2.57	0–51
Age, Class 2	155	85.8 (78.72–90.90)	20.3 ± 2.62	133	23.7 ± 2.95	0–227
Sex, males	115	63.5 (56.04–70.39)	9.1 ± 1.23	73	14.3 ± 1.65	0–62
Sex, females	126	77.0 (69.75–83.04)	20.3 ± 3.20	97	26.4 ± 3.95	0–227
Combined Data	241	70.5 (66.01–74.72)	14.9 ± 1.80	170	21.2 ± 2.40	0–227

Abundance is the mean worm burden (±S.E.M.) for all mice, including uninfected individuals, while intensity is the mean worm burden (±S.E.M.) for mice with at least one worm. The 95% confidence limits for prevalence are provided in parenthesis.

Nevertheless, it was of interest to look closely at how the infection metrics vary between the two age classes and between the sexes, and in the case of mature female mice, whether they differed between mice that were or were not pregnant. The salient details are summarized in Table 2. These show clearly that while prevalence was similar among mature males and females, values for abundance, maximum worm burden, biomass and intensity were all higher for mature females compared to the other data subsets, irrespective of whether the mice were pregnant or not. Neither worm biomass nor abundance differed between pregnant and non-pregnant mature female mice (Mann-Whitney *U* test, sample sizes as in Table 2, for worm biomass *U* = 322.0, *P* = 0.89; for abundance *U* = 444.0, *P* = 0.52).

**Table 2.** Summary statistics for *Protospirura muricola* in juvenile and mature, male and female mice, and in pregnant and non-pregnant females Table 2 long description.

Data subset	Prevalence	Abundance	Range	Biomass (mg).	Range (mg.)	Intensity
	(*n*)	(*n*)		(*n*)	(*n*)	(*n*)
Juvenile males	32.6 (18.52–49.37)	3.8 ± 1.54	0-46	1.1 ± 0.77	0-27.6	11.7 ± 4.07
	(43)	(43)		(37)	(37)	(14)
Juvenile females	53.5 (36.49–69.20)	6.6 ± 2.02	0-51	50.4 ± 25.7	0-689.2	12.3 ± 3.37
	(43)	(43)		(31)	(31)	(23)
Mature males	81.9 (71.28–89.43)	12.2 ± 1.63	0-62	87.2 ± 24.81	0-1144.3	14.9 ± 1.81
	(72)	(72)		(61)	(61)	(59)
Mature females	89.2 (78.73–95.00)	27.4 ± 4.56	0-227	132.9 ± 31.12	0-1145.7	30.7 ± 4.98
	(83)	(83)		(68)	(68)	(74)
Pregnant mature females	90.9 (59.56–99.53)	25.5 ± 7.23	0-65	106.8 ± 55.44	0-570.5	28.0 ± 7.48
	(11)	(11)		(11)	(11)	(10)
Non-pregnant mature females	88.9 (79.29–94.55)	27.7 ± 5.15	0-227	138.0 ± 35.68	0-1145.7	31.1 ± 5.65
	(72)	(72)		(57)	(57)	(64)

For a further break-down of these data by host intrinsic (age and sex) and extrinsic factors (site and year), in association also of two additional wadis, see Behnke et al. (2019).

### Frequency distributions of worm burdens by number of worms recovered

**Figure 1.**
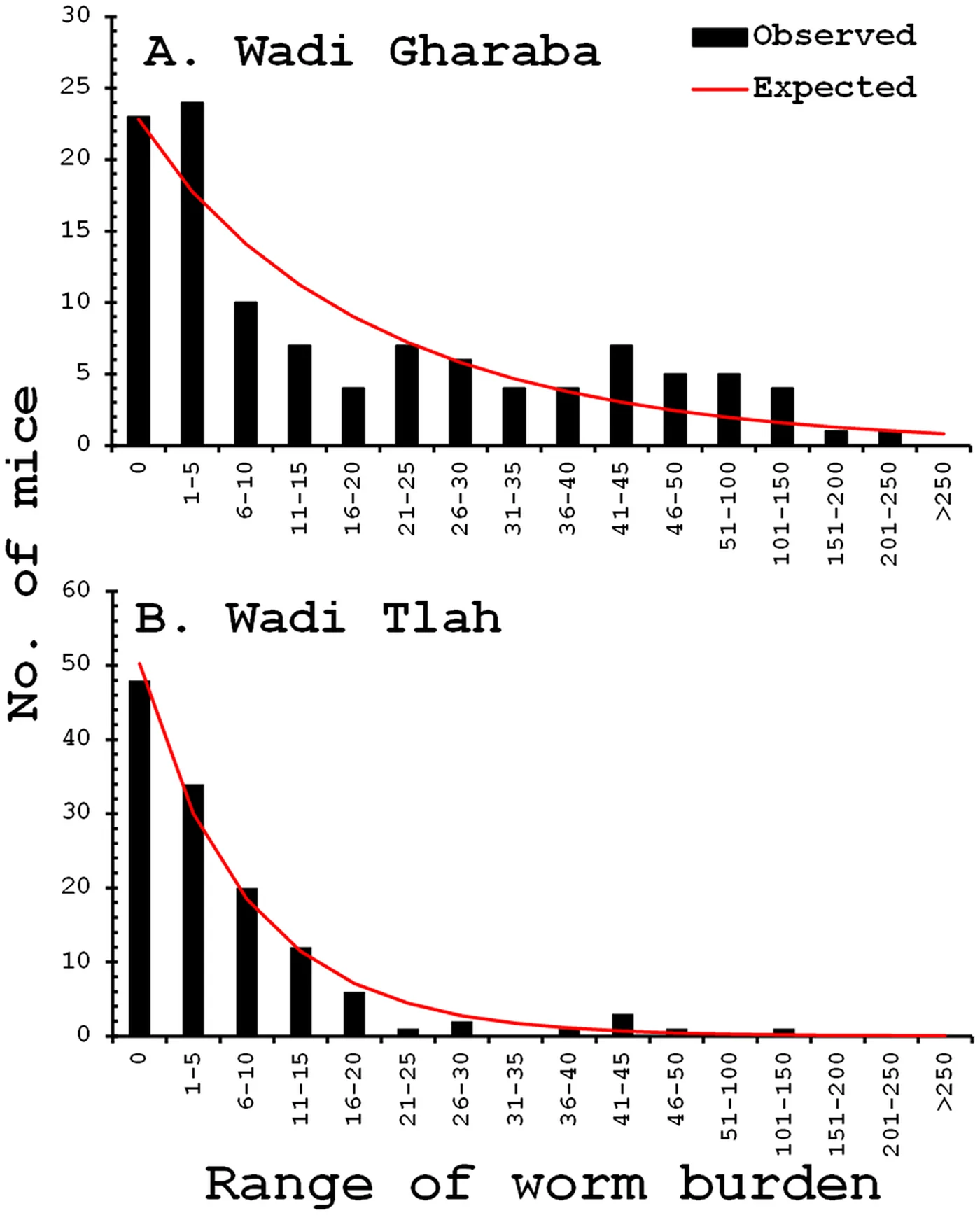
Frequency distributions of worm burdens of *Protospirura muricola* in (A) Wadi Gharaba and (B) Wadi Tlah. Note that the intervals on the abscissa (x-axis) are in classes of 5 between 0 and 50 and in classes of 50 for higher values. Values expected for a negative binomial distribution are shown via red lines. For the goodness of fit to the negative binomial distribution of worm burdens of mice from Wadi Gharaba *χ*^2^_7_ = 12.3, *P* = 0.092, and for those from Wadi Tlah *χ*^2^_5_ = 1.6, *P* = 0.904. The distribution of frequencies differed significantly between the two sites (Anderson-Darling *k*-sample test [*n*_1_ = 16 and *n*_2_ = 16], *AD* = 2.8, *P* = 0.0306). For measures of aggregation, see Table 3. Figure 1 long description.

The data illustrated in Figure 1 and the associated measures of aggregation (Table 3) show that in both wadis (Gharaba and Tlah) the worm burdens of *P. muricola* among the sampled mice conformed well to the negative binomial distribution. This is perhaps best emphasized by the values of I (the variance/mean ratio), which in the case of Wadi Gharaba exceeded 50 and Wadi Tlah 30. In Wadi Gharaba, 17.9% of the mice harboured 60.25% of the worms, while in Wadi Tlah, 12.4% of the mice harboured 59.7% of the worms. The highest worm burden in Wadi Gharaba was 227 (Table 1), and six mice carried more than 100 worms. In Wadi Tlah the highest worm burden was 143, and this was the only mouse with a worm burden exceeding 100. The distribution of worm burdens differed significantly between the spiny mice from Wadis Gharaba and Tlah as illustrated in Figure 1 and in the accompanying statistical analyses (legend for Figure 1). There were more heavily infected mice in Wadi Gharaba, compared with Wadi Tlah, and more uninfected mice in Wadi Tlah compared with Wadi Gharaba.

**Table 3.** Measures of aggregation Table 3 long description.

Data subset	*I*^a^	*D*^b^	*k* ± SEM^c^	No. (%) mice with ≈60.0% worms
Wadi Gharaba	53.65	0.647	0.417 ± 0.0030	20 (17.9%) mice carried 60.25% worms
Wadi Tlah	32.53	0.719	0.321 ± 0.0021	16 (12.4%) mice carried 59.7% worms
Year 2000	39.31	0.668	0.326 ± 0.0070	5 (15.6%) mice carried 58.6% worms
Year 2004	69.78	0.699	0.377 ± 0.0050	7 (11.9%) mice carried 58.4% worms
Year 2008	44.08	0.717	0.275 ± 0.0026	8 (12.1%) mice carried 58.5% worms
Year 2012	53.49	0.721	0.334 ± 0.0028	10 (11.9% mice carried 59.7% worms
Age, Class 1	26.77	0.825	0.158 ± 0.0009	6 (7.0%) mice carried 59.6% worms
Age, Class 2	52.27	0.648	0.526 ± 0.0034	25 (16.1%) mice carried 60.3% worms
Sex, males	19.09	0.679	0.329 ± 0.0024	17 (14.8%) mice carried 59.3% worms
Sex, females	63.45	0.711	0.353 ± 0.0019	17 (13.5%) mice carried 60.5% worms
Combined data	52.50	0.720	0.324 ± 0.0009	31 (12.9%) mice carried 60.2% worms

a *I* = variance/mean ratio.

b *D* = index of dispersion. See (Poulin, 1993).

c *k* = negative binomial exponent.

For sample sizes, see Table 1.

### Variation in frequency distributions of worm burdens between surveys

**Figure 2.**
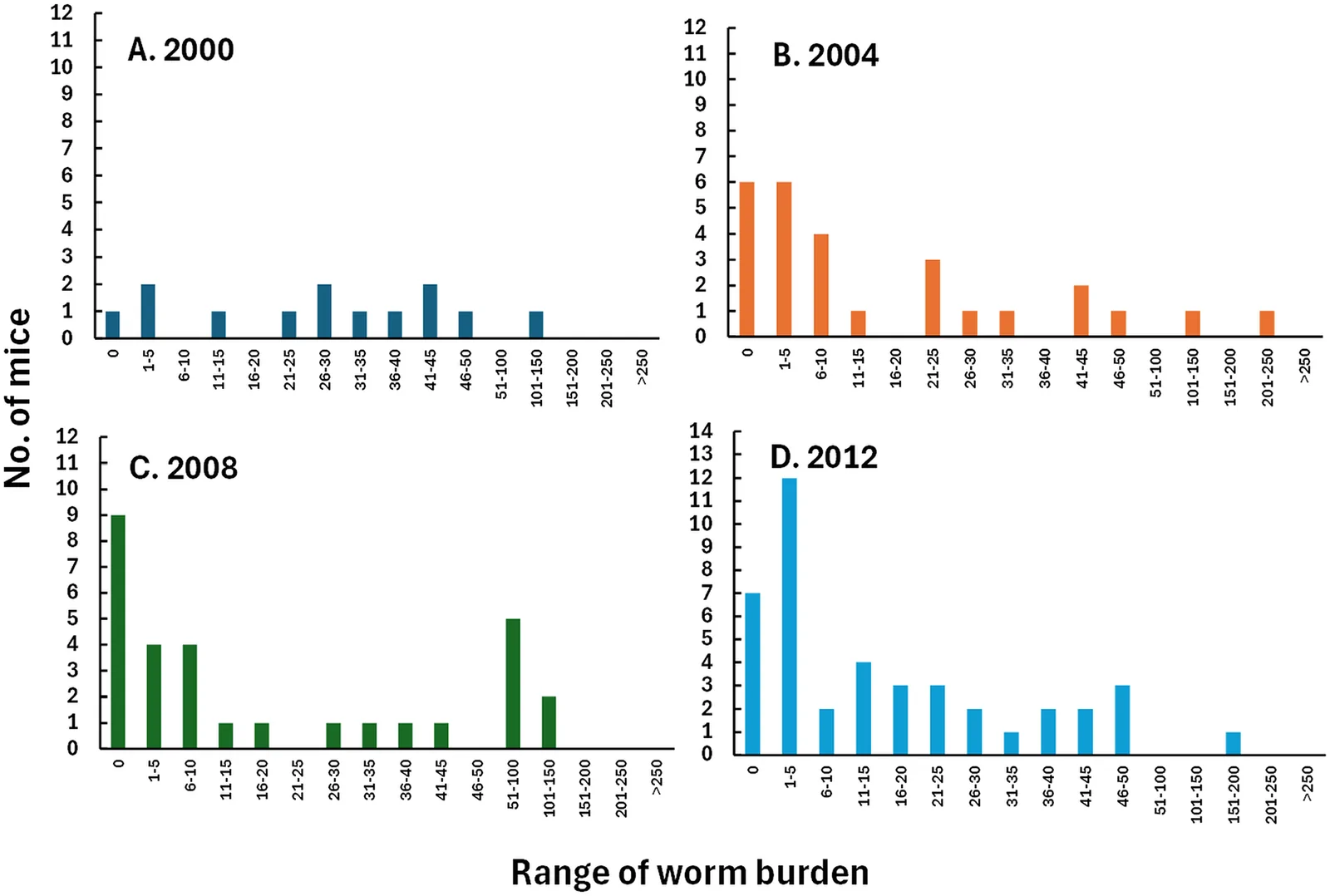
Frequency distribution of *Protospirura muricola* in Wadi Gharaba by year of survey. The distribution of frequencies across the four surveys did not differ significantly between years (Anderson-Darling *k*-sample test [*n*_1_, *n*_2_, *n*_3_ and *n*_4_ = 16 each, *AD* = 3.360, *P* = 0.309). For measures of aggregation, see Table 3. Figure 2 long description.

**Figure 3.**
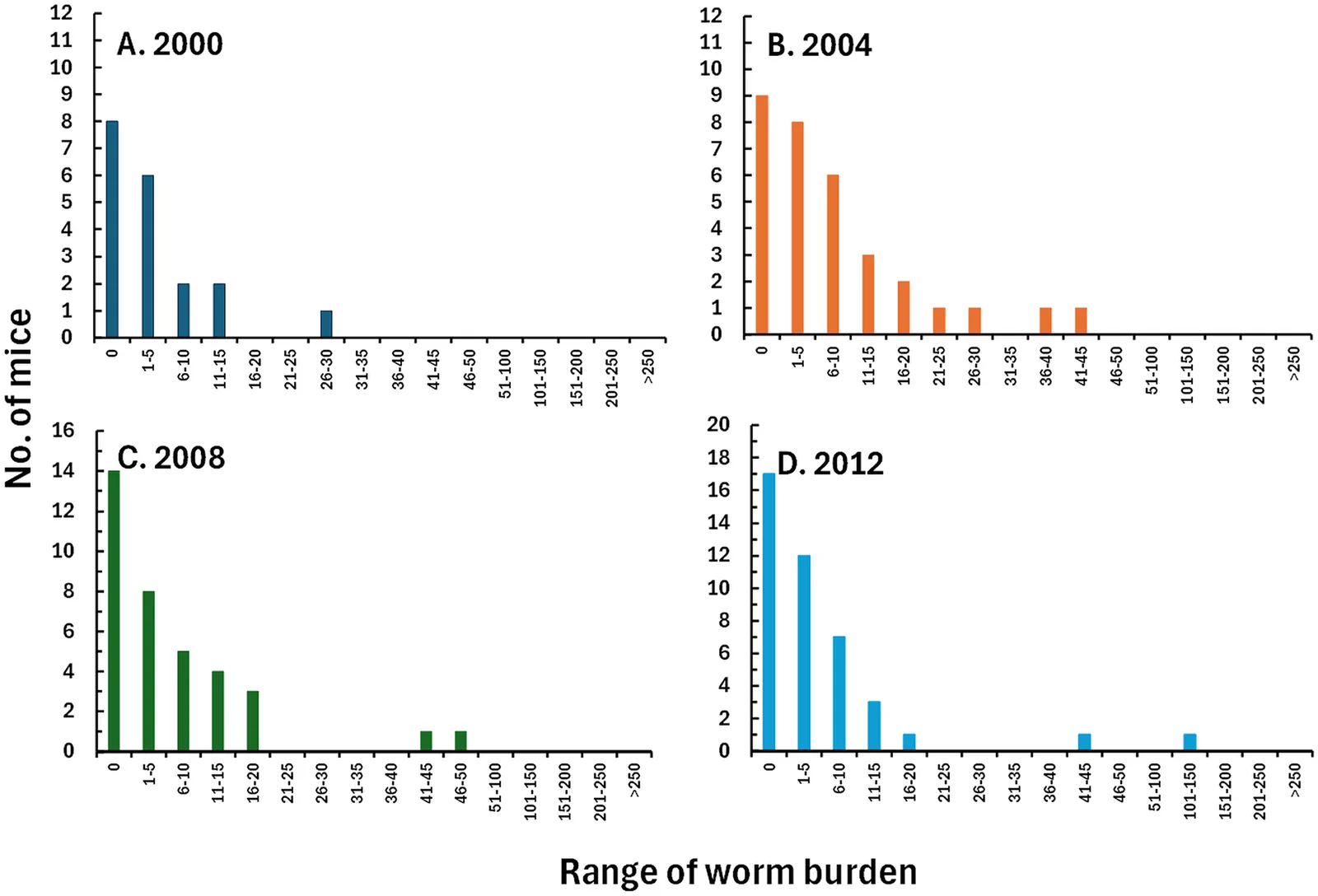
Frequency distribution of *Protospirura muricola* in Wadi Tlah by year of survey. The distribution of frequencies across the four surveys did not differ significantly between years (Anderson-Darling *k*-sample test [*n*_1_, *n*_2_, *n*_3_ and *n*_4_ = 16 each, *AD* = 1.650, *P* = 0.906). For measures of aggregation, see Table 3. Figure 3 long description.

There was no significant difference between surveys in the frequency distribution of worm burdens among the sampled mice from Wadi Gharaba (Figure 2, statistical analysis shown in Figure 2 legend and Table 3), nor from Wadi Tlah (Figure 3, statistical analysis in Figure 3 legend and Table 3).

### Variation in frequency distributions of worm burdens between the sexes

**Figure 4.**
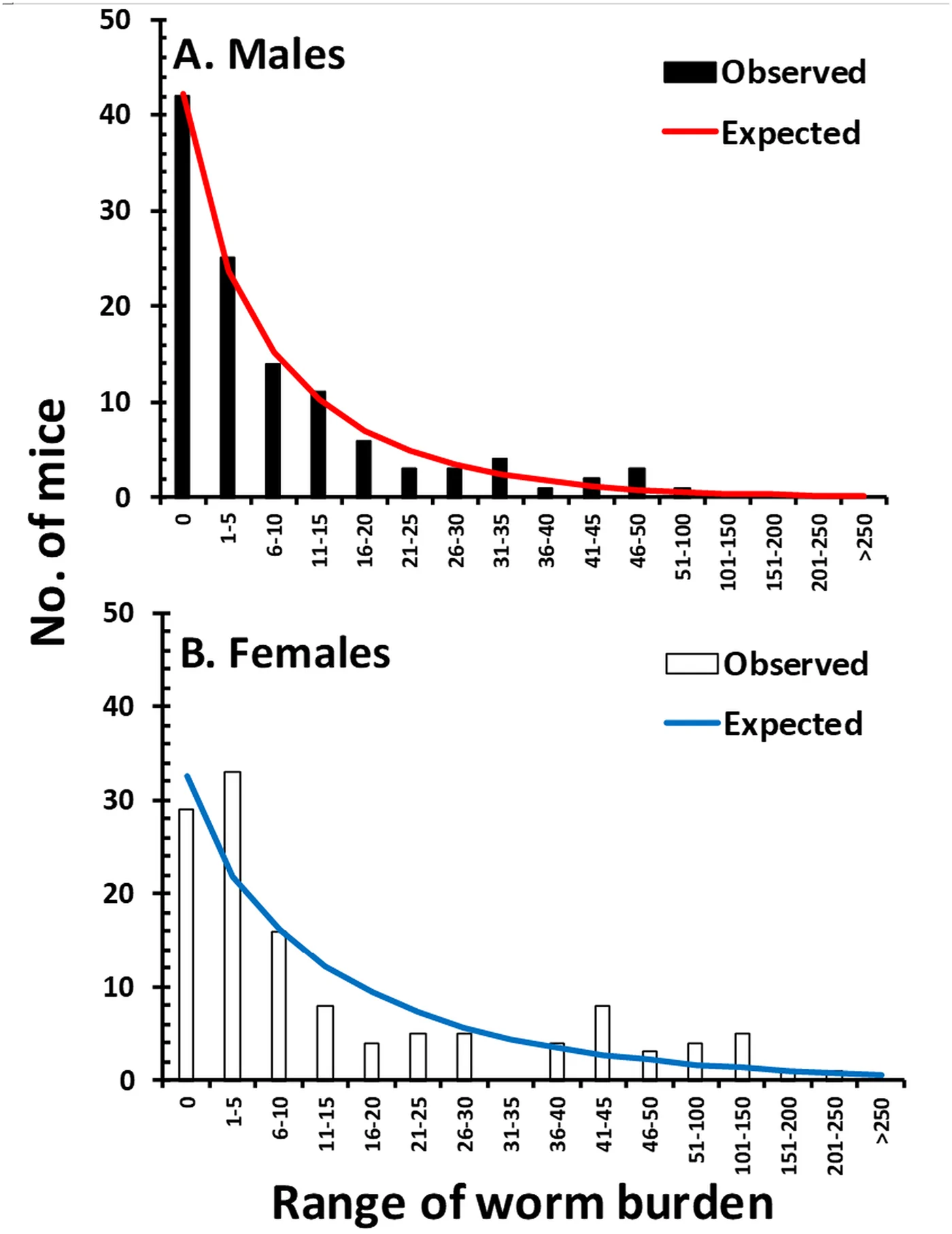
Frequency distribution of *Protospirura muricola* in male and female mice. Combined data from all four surveys and both wadis. Note that the intervals on the abscissa (x-axis) are in classes of 5 between 0 and 50 and in classes of 50 for higher values. Values expected for a negative binomial distribution are shown via red and blue lines. For the goodness of fit to the negative binomial distribution of worm burdens from male mice *χ*^2^_5_ = 0.4, *P* = 0.995 and for those from female mice *χ*^2^_7_ = 14.0, *P* = 0.051. The distribution of frequencies did not differ significantly between male and female mice (Anderson-Darling *k*-sample test [*n*_1_ and *n*_2_ = 16 each, *AD* = 0.922, *P* = 0.404). For measures of aggregation, see Table 3. Figure 4 long description.

There was no significant difference between the frequency distribution of worm burdens from male and female mice (Figure 4, statistical analysis shown in Figure 4 legend and Table 3). Although the values of *k* and *D* were very similar for both sexes, *I* was much greater for female mice compared to males, reflecting the extreme high values for worm burdens in some of the female mice (Tables 1 and 2). Overdispersion of worm burdens is clearly evident in the high values of *I* and *D* in both sexes and in male mice by the goodness of fit of the data to the expected negative binomial distribution. For female mice, and as illustrated in Figure 4, the fit was only just within the non-significant category. There were several cases of mismatch between observed and expected numbers of mice, as for example among those in harbouring 1–5 worms and 41–45, and 101–150 worms. While the highest worm burden recorded in male mice was 62 worms, 9 female mice harboured greater worm burdens, 6 between 100 and 200 worms, and 1 harbouring 227 worms.

### Variation in frequency distributions of worm burdens between the age classes

**Figure 5.**
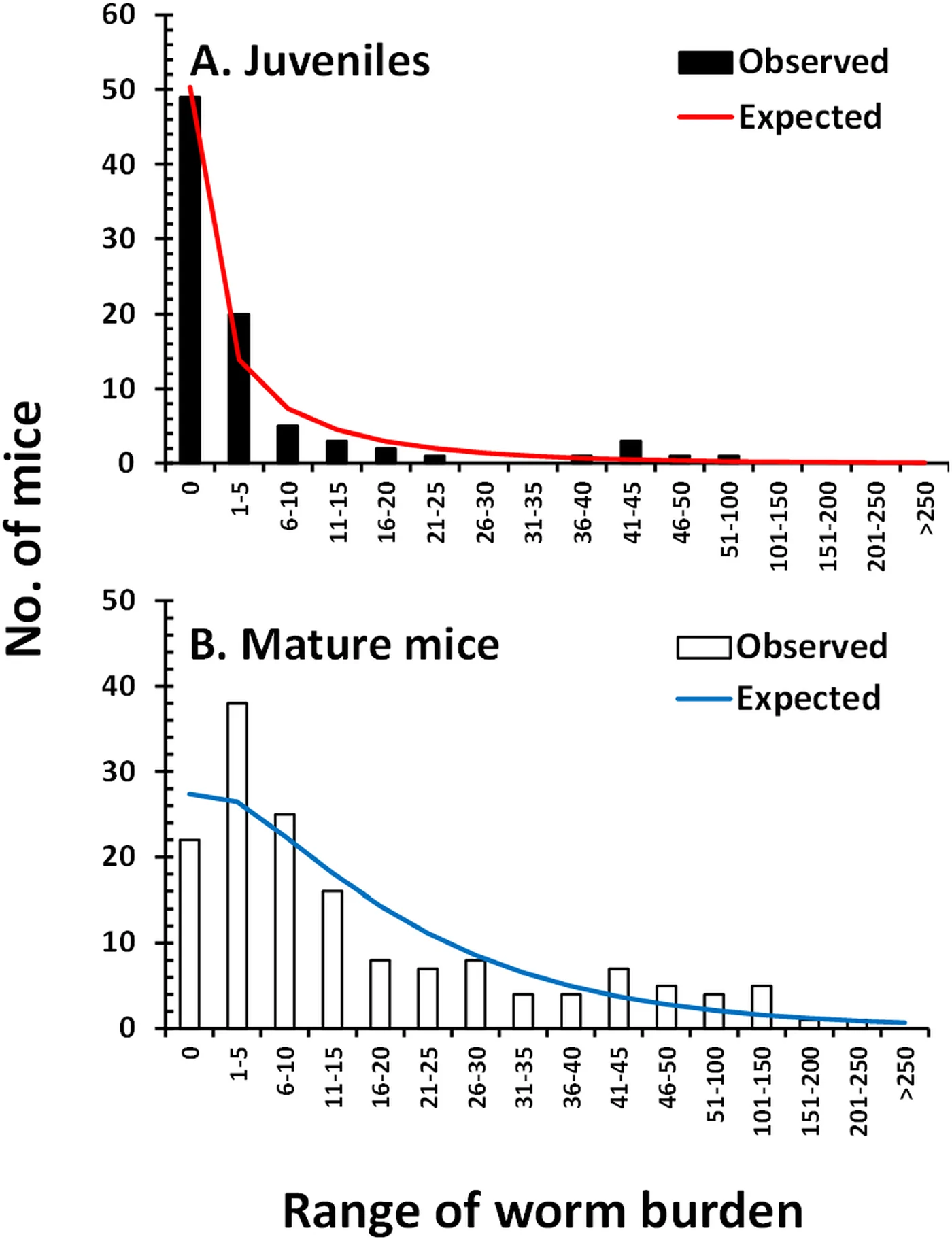
Frequency distribution of *Protospirura muricola* in juvenile and mature mice. Combined data from all four surveys and both sexes. Note that the intervals on the abscissa (x-axis) are in classes of 5 between 0 and 50 and in classes of 50 for higher values. Values expected for a negative binomial distribution are shown via red and blue lines. For the goodness of fit to the negative binomial distribution of worm burdens from juvenile mice *χ*^2^_3_ = 3.8, *P* = 0.289, and for those from mature mice *χ*^2^_8_ = 15.8, *P* = 0.045. The distribution of frequencies differed significantly between juvenile and mature mice (Anderson-Darling *k*-sample test [*n*_1_ and *n*_2_ = 16 each, *AD* = 5.020, *P* = 0.0021). For measures of aggregation, see Table 3.

The frequency distribution of worm burdens in juvenile and mature mice (Figure 5, statistical analysis shown in Figure 5 legend and Table 3) differed significantly. Both the values of *I* and *D* were indicative of highly overdispersed data. The frequency distribution of worms in juvenile mice did not differ significantly from the expected negative binomial distribution, although the test was limited to just 3 degrees of freedom because of the generally low values. For mature mice, the negative binomial distribution was the best fit, although statistically it differed significantly, although marginally, from that of the observed worm burden classes. There were more than expected mice carrying 1–5 worms and at the other end of the range, more carrying 41–150 worms than expected. While the highest worm burden recorded in juvenile mice was 51 worms, 11 mature mice harboured greater worm burdens, 1 harbouring 227 worms.

### Frequency distributions of worm biomass by number of worms recovered

**Figure 6.**
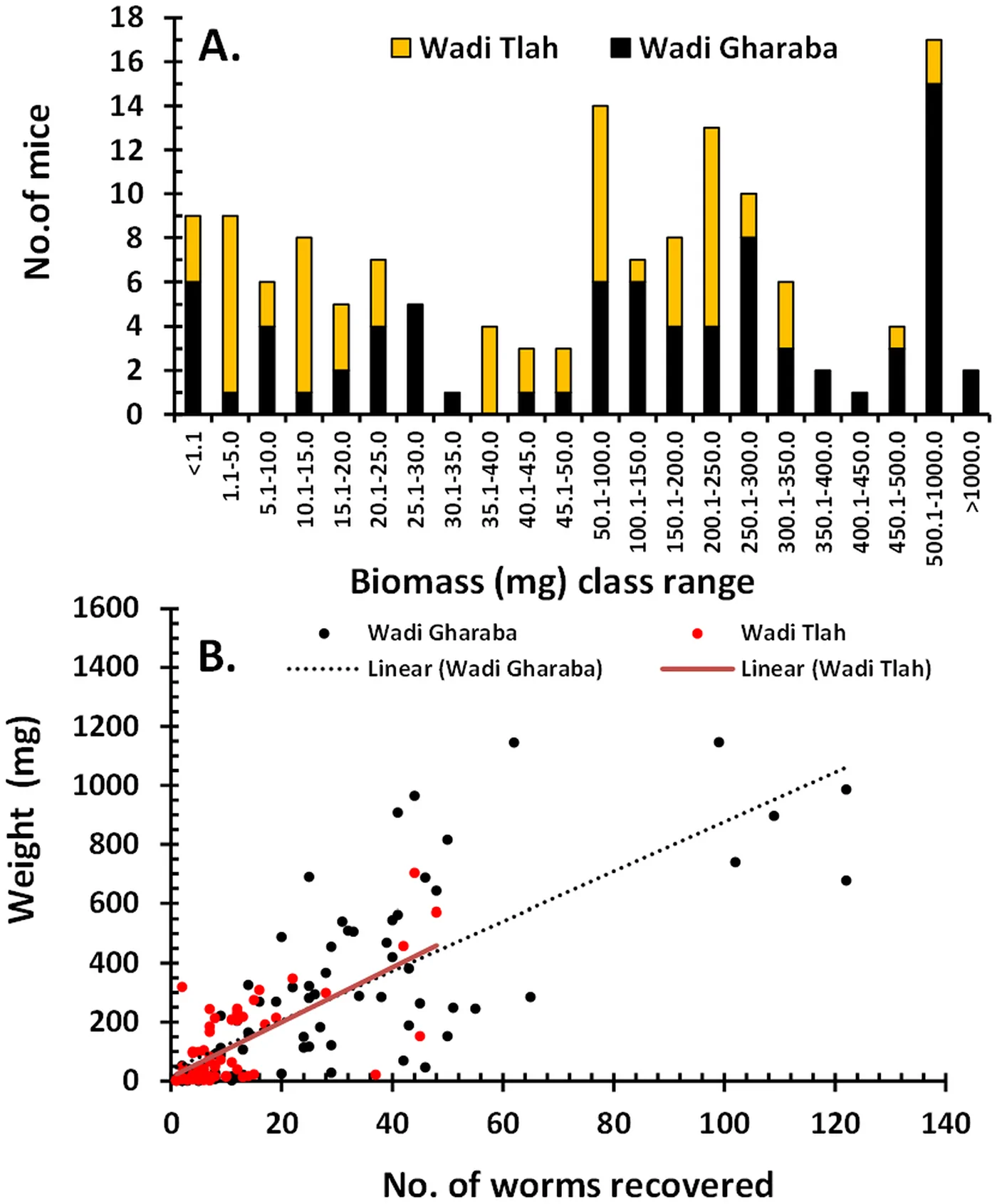
Relationships between total worm biomass and worm burdens. (A) Frequency distribution of worm biomasses by worm burden class. Note the discontinuous scale on the abscissa to accommodate the range of worm burdens, with worm burden classes of 5 in the range from 1 to 50, worm burden classes of 50 from 50.1 to 500, and 1000 above 500.1. (B) Relationship between the number of worms recovered and the wet worm biomass. The equations for the fitted linear lines are *y* = 13.804 + 9.3063*x* (*R*^2^ = 0.4933) for Wadi Tlah and y = 38.177 + 8.3853x (*R*^2^ = 0.5863) for Wadi Gharaba. Note, two outliers were removed from this dataset, one each from the two wadis both trapped in 2012, because in both cases over 100 larvae that could not be weighed dominated their worm burdens. Mouse 232, with a worm biomass of 258.8 mg from Wadi Gharaba, carried 176 worms, of which 140 were very small larvae. Mouse 249, with worm biomass of 14 mg from Wadi Tlah, carried 143 worms of which 142 were too small to weigh accurately. Figure 6 long description.

The biomass of *P. muricola* carried by individual mice varied from 0.1 mg to 1145.7 mg. Two mice from Wadi Gharaba carried biomasses of over 1 gm, one a male and the other a female, both mature adult animals. For the female mouse weighing 37.8 g, this represented 3.0% of its total body weight. The male mouse carried a biomass of 1144.3 mg and weighed 40.1 g, the worm biomass therefore representing 2.85% of total body weight. Seventeen mice carried spirurid biomasses of 500−1000 mg and 51 in the range 100–500 mg. More detailed subdivision of spirurid biomass values is illustrated in Figure 6A. Although mice from both wadis contributed to most of the biomass classes, only three mice from Wadi Tlah carried biomasses over 350 mg.

### Variation in worm burdens and worm biomass with increasing host age

**Figure 7.**
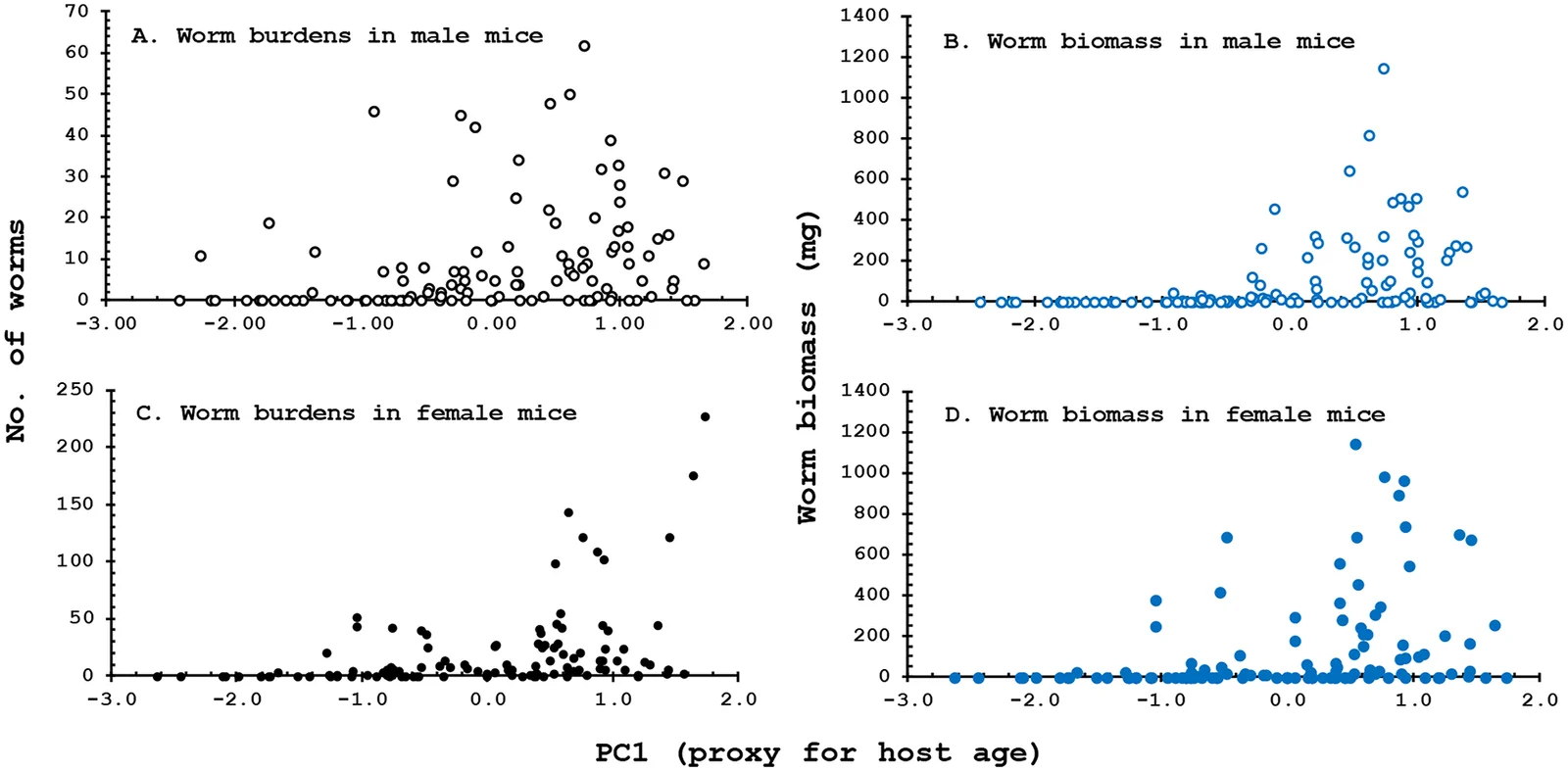
Covariance of *Protospirura muricola* worm burdens (A and C) and worm biomasses (B and D) with PC1 (proxy for host age) in male (A and B) and female (C and D) mice. In A, *R_s_* = 0.422, *n* = 114, *P* < 0.001; B, *R_s_* = 0.688, *n* = 98, *P* < 0.001; C, *R_s_* = 0.505, *n* = 111, *P* < 0.001; D, *R_s_* = 0.578, *n* = 86, *P* < 0.001. Figure 7 long description.

The summary statistics for host age in Tables 1, **2** and 3 are based on two age classes, with hosts allocated to each class as described in Behnke et al. (2019). As can be seen, there is a very large difference in both prevalence and abundance of *P. muricola* between juvenile and mature mice, and the frequency distributions indicate that heavy worm burdens are more likely to occur among mature, rather than juvenile mice. However, to generate a more nuanced picture of how worm burdens increase with increasing host age, we used PC1s (as proxies for age) from PCAs conducted separately on each sex. We also conducted a similar exercise using worm biomass. While uninfected mice occurred across the whole range of PC1, heavy infections and mice carrying large biomasses of *P. muricola* were more likely to occur among the older mice in both sexes (Figure 7), but especially in female mice. For worm burdens and worm biomasses in both sexes, there was a highly significant positive covariance with PC1 (see Figure 7 legend for statistical parameters).

### Relationship between worm biomass and worm burden

Covariance of spirurid biomass and the total number of worms recovered (Figure 6B) was highly significant (*t*_142_ = 14.98, *P* < 0.00001 and *n* = 144, *R* = 0.783 [CL_95_ = 0.710–0.839]). Highly significant covariance was also evident when the data from each wadi were analysed separately (Wadi Gharaba *n* = 80, *R* = 0.766 [CL_95_ = 0.656-0.844], and Wadi Tlah *n* = 64, *R* = 0.702 [CL_95_ = 0.552-0.809]), and the difference in covariance between the wadis was not significant (2-way interaction for no. of worms x site, on worm biomass, *F*_1, 140_ = 0.22, *P* = 0.640).

### Frequency distributions of worm weights in individual mice

**Figure 8.**
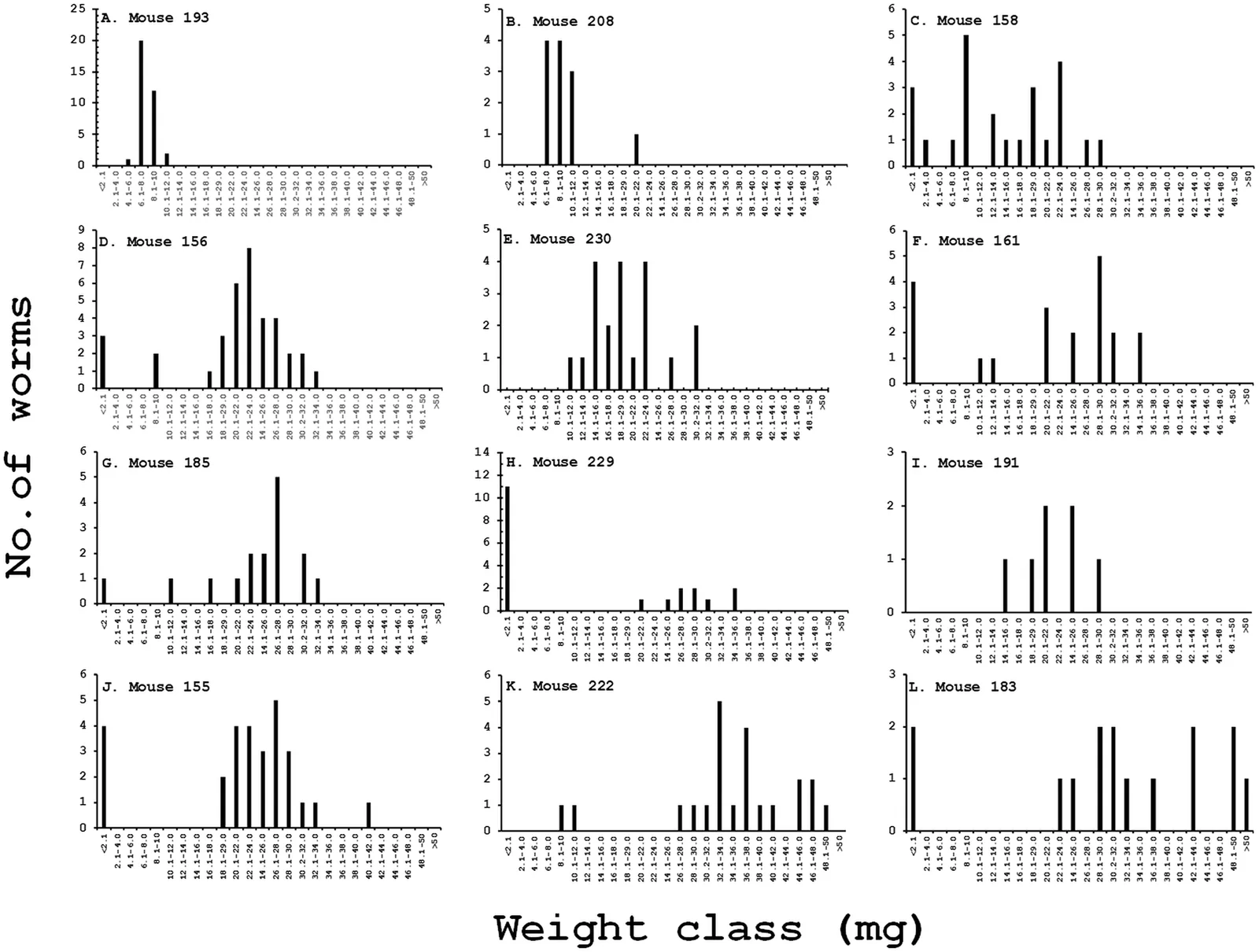
Frequency distributions of worms by worm weight classes (2 mg classes) from 12 selected mice from the survey in 2012. Each panel corresponds to one mouse and the mouse reference is given in the top left-hand corner of each panel. Figure 8 long description.

In comparison to many other intestinal nematode parasites of rodents, *P. muricola* has a relatively long development time following infection with insect-borne L3 to patency in its definitive hosts. Over the course of 40–60 days post infection, female worms grow from a length of 2–3 mm to over 5 cm (Gedoelst, 1916; Tubangui, 1931; Quentin, 1969; Foster and Johnson, 1939, recorded adult females of 6.5–7.0 cm in length in captive white-faced monkeys) and from a weight of <2 mg for infective larvae to achieve a final weight of 30–60 mg (Figure 8; Lowrie et al., 2004). In ecosystems where transmission is high and frequent, this long development time results in hosts harbouring a range of worms differing in length and weight, corresponding to individual transmission events.

We examined the frequency distribution of weighed individual worms in mice which carried some of the heavier worm burdens, from the survey in 2012. As can be seen from Figure 8, some mice carried worms of quite different weights, each weight class represented by just a few worms. In others the worm weights were clearly clumped, forming two or more peaks (Figure 8C, E, F, L). Figure 8, panel A, shows the frequency distribution of worms by weight in a young male mouse which most likely acquired 20 or more worms from a single infected insect host. Other mice showed a large range of worm weights, with peaks mostly fewer than 5 worms, indicating multiple low-dose transmission (e.g. Figure 8C–F, J, K). Older mice (Figure 8K, L) harboured the heavier worms but lacked worms with intermediate weights, although in some cases were still susceptible to infection with very small, presumably recently acquired larvae (e.g. Figure 8K, L). Two adult mice (not illustrated in Figure 8) were found to harbour heavy infections, that comprised just a few adult worms, but high numbers of very small larvae (mouse 232 from Wadi Gharaba carried 176 worms, of which 140 were very small larvae and mouse 249 from Wadi Tlah, carried 143 worms of which 142 were too small to weigh accurately), suggesting in both cases recent consumption of heavily infected insect hosts.

## Discussion

*Protospirura muricola* is a relatively understudied species, given its wide distribution in rodents across the tropics and subtropics. Analyses of our long-term data from two wadis in the Sinai of Egypt, where prevalence was particularly high, have therefore made a substantial contribution to our understanding of the epidemiology of this species. Like many other helminths, *P. muricola* shows an aggregated distribution in its host population, with high intensity worm burdens restricted to relatively few mature mice, but especially, although not exclusively, to the older females. Remarkably, while the frequency distribution of worm burdens among the host populations differed significantly between the mice from the 2 wadis, it did not change significantly between years in either wadi. This is consistent with the summary metrics also reported here, which show stability in prevalence and abundance of the species in the four surveys spaced at 4-year intervals over a period of 12 years. Our earlier analysis, which included two additional wadis, also found that prevalence and abundance of *P. muricola* did not vary significantly between surveys (Behnke et al., 2019).

Much as expected, the frequency distribution of worm burdens differed significantly between juvenile and mature mice, and a more nuanced approach exploiting a continuous range for host age (rather than a binomial factor), clarified that heavier worm burdens were considerably more likely in older animals of both sexes, but especially among female mice. To a large extent, the higher overall prevalence and heavier worm burdens in older female mice contributed to the significant difference in abundance between the sexes. Although prevalence and abundance of intestinal helminths have been found often to be male-biased in mammalian hosts, female bias is also known (Zuk, 1990; Poulin, 1996; Zuk and McKean, 1996; Moore and Wilson, 2002; Perkins et al., 2008; Berland et al., 2025). The related spirurid nematode *Mastophorus muris*, for example, showed significantly higher prevalence and abundance in female bank voles (*Clethrionomys glareolus*) compared with males (Behnke et al., 2008; Grzybek et al., 2015a). Infection metrics were also higher among lactating voles, and the greater need for higher invertebrate content in the diet of pregnant and nursing female hosts was hypothesized as the most likely explanation (Grzybek et al., 2015b).

Reports of *P. muricola* in the literature are mostly confined to surveys of naturally infected rodents and to taxonomic studies (Baylis, 1928; Scharff et al., 1993; Waugh et al., 2006; Smales et al., 2009). Experimental studies with laboratory-maintained isolates are rare (Lowrie et al., 2004) but among the notable findings of such studies are the very long duration of the survival of viable worms in cysts in beetles (up to 683 days in Schutgens et al., 2015), the long prepatent period and the duration of survival of adult worms in laboratory mice (Lowrie, 2003). *Protospirura muricola* is a slowly growing nematode whose embryonated eggs appear in host faeces approximately 60 days after infection, although a few non-embryonated eggs may be detected from day 40 post infection (Quentin, 1969; Lowrie, 2003). Moreover, the adult worms can live for at least a further 4 months, eggs having been detected in mouse faeces for up to 191 days after an experimental infection with 5 cysts and were still being shed in small numbers by *in vitro* incubated 200 days-old female worms (Lowrie, 2003). Although similar experiments have not been conducted in spiny mice, the epidemiological data support the idea that this is a long-lived parasite, which accumulates in mice with increasing host age (Behnke et al., 2000, 2019). This longevity, slow development, late reproduction, eventual large size of the adult worms and stability of abundance in its host populations are all indicative of *P. muricola* being subject to a K-selection strategy (MacArthur, 1962; Pianka, 1970; Reznick et al., 2002).

The reported intermediate hosts of *P. muricola* include cockroaches of several species (*Leucophaea maderae, Perplaneta americana* and *P. brunnea*; Foster and Johnson, 1939; Campos and Vargas, 1977) and earwigs (Dermaptera; Quentin , 1969). *Blattelia germanica* has been recorded in the village of St. Katherine (F. Gilbert pers. obs.), and we have observed cockroaches in large numbers in Wadi Gharaba, especially concentrated around the Ecolodge (SE Griffiths and L. Elton, pers obs. but not identified to species). Several species of earwigs are known to exist in Egypt (Krauss, 1909; Aboelhadid et al., 2022), and some have been recorded in surveys of insects in Northern Sinai (El-Moursy et al., 2001) as well as locally in St. Katherine's Protectorate in the south of the peninsula (Abdel-Ghany et al., 2007). El-Moursy et al. (2001) recorded the presence of *Labidura* sp., a genus known to be highly susceptible to *P. muricola* (Quentin, 1969). Zalat et al. (2001) found that locally in St Katherine's Protectorate, the highest diversity and species richness of ground insects was in Wadi Gharaba, and among these they recorded several species of Coleoptera, while in a further survey, Zalat et al. (2008) recorded over 50 species of Coleoptera from 19 families in neighbouring wadis in St Katherine's Protectorate. Norfolk et al. (2012) reported that beetles were the most diverse group of arthropods (18 species from 11 families of Coleoptera) in surveys conducted in neighbouring wadis in the Sinai, which included Wadi Tlah. All 3 surveys recorded various species of Tenebrionidae (see also Semida et al., 2001; Lillig and Pavlíček, 2022, for longer comprehensive lists of Coleoptera, including the Tenebrionidae, recorded throughout the Sinai). Although naturally infected Coleoptera have not been recorded in the field, laboratory studies have shown that the tenebrionid beetles *Tribolium confusum* and *Tenebrio molitor* are both susceptible to infection (Schutgens et al., 2015).

The eggs of *P. muricola* are robust and protected by a thick eggshell (see Brumpt, 1931 for description of eggs of the related *P. bonnie*), but perhaps unexpectedly they are already embryonated on passage out of the host in faeces and infective to beetles within 24 h (Lowrie, 2003). Laboratory exposures of the tenebrionid flour beetles (*Tribolium confusum*) to the eggs of *P. muricola* mixed with flour have resulted generally in low level intensity, only 1–2 cysts with an average of 2.9 cysts per beetle, although some beetles have been observed to harbour up to 8 cysts (Schutgens et al., 2015). Larger insects may carry heavier infections. Naturally infected cockroaches (*L. maderae*), for example, have been recorded as carrying several hundred cysts with an average of approximately 100 cysts (Foster and Johnson, 1939). Coprophagic arthropods feeding on rodent faeces are therefore likely to acquire heavy infections, and a mouse preying on a heavily infected insect is likely to acquire a package of infective worms, which should then develop uniformly in the stomach. Therefore, we can expect trophically transmitted helminths exploiting arthropod intermediate hosts to be transmitted by intermittent clumped transmission when their vertebrate hosts predate intermediate hosts that harbour more than just one infective stage of the parasite.

The slow development of *P. muricola* in its definitive hosts and the eventual large size and weight of fully mature worms have allowed worms in hosts harbouring heavy infections to be separated into classes of similar weight, each of which is likely to correspond to the consumption of a single intermediate host. Such a conclusion assumes that there is little or no acquired host-protective immunity to *P. muricola* in its hosts. Nothing is currently known about immunity to this species, but the increasing intensity of worm burdens in hosts of increasing age (Behnke et al., 2019) indicates that it is likely to be weak, if at all. Another possible complication may arise from density-dependent constraints on worm growth. Lowrie et al. (2004) showed that in laboratory mice, there was significant increasing restriction of growth of worms with increasing doses of administered infective larvae, but over a period of 30 days, this was small, accounting for approximately 16.0% of length in female worms and no significant reduction in weight.

Therefore, assuming little or no acquired immunity and some but only modest density dependent constraints on growth, our data have shown that some mice carry a large range of differently sized worms that reflect consumption of many insects carrying single or a few cysts. Others, however, harbour many worms of a similar weight that have not yet reached their expected final weight. These are therefore more likely to represent cases of clumped transmission, reflecting acquisition of multiple worms over a very limited period of time, hence mostly probably from individual insects harbouring many cysts.

The analyses of the frequency distributions of *P. muricola* worm burdens detailed in this paper have shown that like abundance and prevalence (Behnke et al., 2019), this metric of infection was relatively stable over more than a decade among spiny mice in 2 wadis where transmission was intense. We have identified also a subset of the mouse population (mature older female mice) that was particularly at risk of acquiring heavy parasite burdens and shown that transmission among these hosts likely involves frequent low-dose acquisition of worms from insect vectors, with less frequent package transmission involving heavily infected intermediate hosts. The identity of the exact insects involved in transmission in the region remains to be ascertained but inevitably is likely to involve ground-dwelling species such as the Coleoptera, Dermaptera and Dictyoptera (Blattidae), some of which abound in the 2 wadis where the spiny mice were captured.

## Data Availability

The data employed in the analyses reported in this paper can be made available from the corresponding author on reasonable request.

## Table 1 Long description

Protospirura muricola infection metrics are summarized for mice by site, year, age class, sex, and overall, reporting sample size, prevalence with 95 percent confidence limits, mean abundance with standard error, number infected, mean intensity with standard error, and worm-count range. By site, Wadi Gharaba shows higher prevalence (79.5 percent) and higher abundance (23.8 worms on average across all mice) than Wadi Tlah (62.8 percent prevalence; 7.3 abundance). Across years, prevalence stays broadly similar (about 65 to 75 percent), while abundance ranges from 13.4 to 15.9 and intensity from 18.8 to 24.0, with the highest maximum worm count reported in 2004 (range up to 227). Age class differs strongly: Class 2 has much higher prevalence (85.8 percent) and abundance (20.3) than Class 1 (43.0 percent prevalence; 5.2 abundance). Females show higher prevalence (77.0 percent) and abundance (20.3) than males (63.5 percent prevalence; 9.1 abundance), and higher intensity among infected mice (26.4 versus 14.3). Overall combined results are 241 mice with 70.5 percent prevalence, 14.9 mean abundance, 170 infected, 21.2 mean intensity, and a range from zero to 227 worms. Abundance includes uninfected mice, so it is lower than intensity and should not be compared as if it reflects only infected individuals.

Navigate back to Table 1.

## Table 2 Long description

Protospirura muricola infection metrics are reported for juvenile and mature mice by sex, plus pregnant and non-pregnant mature females. Measures include prevalence with confidence intervals, mean abundance with spread, abundance range, mean biomass in milligrams with spread, biomass range, and mean intensity with spread; sample sizes vary by metric. Prevalence rises from 32.6 percent in juvenile males and 53.5 percent in juvenile females to 81.9 percent in mature males and 89.2 percent in mature females. Mean abundance increases with age and is highest in mature females at 27.4, compared with 12.2 in mature males, 6.6 in juvenile females, and 3.8 in juvenile males. Intensity follows the same pattern, reaching 30.7 in mature females versus 14.9 in mature males and about 12 in both juvenile groups. Biomass is much higher in mature groups, with mean biomass 132.9 milligrams in mature females and 87.2 milligrams in mature males; juvenile males average 1.1 milligrams while juvenile females average 50.4 milligrams, with very wide biomass ranges in females. Pregnant mature females have prevalence 90.9 percent and slightly lower mean abundance, biomass, and intensity than non-pregnant mature females, but the pregnant subgroup is small, so differences should be interpreted cautiously.

Navigate back to Table 2.

## Figure 1 Long description

Bar graph titled Wadi Gharaba with a legend listing Observed and Expected. The horizontal axis label is Range of worm burden. The vertical axis label is No. of mice. The vertical axis shows labeled values 0, 5, 10, 15, 20, 25, 30. The horizontal axis categories are 0, 1 to 5, 6 to 10, 11 to 15, 16 to 20, 21 to 25, 26 to 30, 31 to 35, 36 to 40, 41 to 45, 46 to 50, 51 to 100, 101 to 150, 151 to 200, 201 to 250, greater than 250. Observed is shown as vertical bars and Expected is shown as a smooth curve. The tallest observed bar is at category 0, with the bar height between 20 and 25 on the No. of mice scale. Observed bars decrease across the next categories, with smaller bars through 1 to 5, 6 to 10, 11 to 15, 16 to 20, 21 to 25, 26 to 30, 31 to 35, 36 to 40, 41 to 45 and 46 to 50. Observed bars are also present in higher ranges 51 to 100, 101 to 150, 151 to 200 and 201 to 250, with bar heights close to the lower part of the scale. The Expected curve starts near the upper part of the scale at the left and declines steadily toward the right, approaching the baseline by the highest burden categories. Bar graph titled Wadi Tlah with a legend listing Observed and Expected. The horizontal axis label is Range of worm burden. The vertical axis label is No. of mice. The vertical axis shows labeled values 0, 10, 20, 30, 40, 50, 60. The horizontal axis categories are 0, 1 to 5, 6 to 10, 11 to 15, 16 to 20, 21 to 25, 26 to 30, 31 to 35, 36 to 40, 41 to 45, 46 to 50, 51 to 100, 101 to 150, 151 to 200, 201 to 250, greater than 250. Observed is shown as vertical bars and Expected is shown as a smooth curve. The tallest observed bar is at category 0, with the bar height between 40 and 50 on the No. of mice scale. Observed bars drop across 1 to 5, 6 to 10, 11 to 15 and 16 to 20 and continue as smaller bars through 21 to 25, 26 to 30, 31 to 35, 36 to 40, 41 to 45 and 46 to 50. The Expected curve starts near the upper part of the scale at the left and declines toward the right, approaching the baseline by the higher burden categories. Across both graphs, observed counts are highest at the lowest worm burden category and decrease as the worm burden range increases, while the expected curve declines across the same categories. The Wadi Tlah graph uses a higher No. of mice scale (0 to 60) than the Wadi Gharaba graph (0 to 30) and the tallest observed bar at category 0 is higher in Wadi Tlah than in Wadi Gharaba. Observed bars extend into the higher burden ranges 51 to 100 through 201 to 250 in Wadi Gharaba, while Wadi Tlah shows observed bars only up to 46 to 50.

Navigate back to Figure 1.

## Figure 2 Long description

2000. The y-axis label is No. of mice. The y-axis range is 0 to 12. The x-axis label is Range of worm burden. The x-axis categories are: 0, 1 to 2, 3 to 4, 5 to 6, 7 to 8, 9 to 10, 11 to 15, 16 to 20, 21 to 25, 26 to 30, 31 to 35, 36 to 40, 41 to 45, 46 to 50, 51 to 100, 101 to 150, 151 to 200, 201 to 250, greater than 250. Bars shown at: 1 to 2 equals 2; 3 to 4 equals 1; 21 to 25 equals 1; 26 to 30 equals 2; 31 to 35 equals 1; 36 to 40 equals 1; 41 to 45 equals 2; 46 to 50 equals 1; 51 to 100 equals 2; 101 to 150 equals 1. Highest bar values are 2 at 1 to 2, 26 to 30, 41 to 45 and 51 to 100. 2004. The y-axis label is No. of mice. The y-axis range is 0 to 12. The x-axis label is Range of worm burden. The x-axis categories are: 0, 1 to 2, 3 to 4, 5 to 6, 7 to 8, 9 to 10, 11 to 15, 16 to 20, 21 to 25, 26 to 30, 31 to 35, 36 to 40, 41 to 45, 46 to 50, 51 to 100, 101 to 150, 151 to 200, 201 to 250, greater than 250. Bars shown at: 0 equals 6; 1 to 2 equals 6; 3 to 4 equals 4; 5 to 6 equals 1; 26 to 30 equals 3; 31 to 35 equals 1; 36 to 40 equals 1; 51 to 100 equals 2; 101 to 150 equals 1; 151 to 200 equals 1; 201 to 250 equals 1. Highest bar values are 6 at 0 and 1 to 2. 2008. The y-axis label is No. of mice. The y-axis range is 0 to 12. The x-axis label is Range of worm burden. The x-axis categories are: 0, 1 to 2, 3 to 4, 5 to 6, 7 to 8, 9 to 10, 11 to 15, 16 to 20, 21 to 25, 26 to 30, 31 to 35, 36 to 40, 41 to 45, 46 to 50, 51 to 100, 101 to 150, 151 to 200, 201 to 250, greater than 250. Bars shown at: 0 equals 9; 1 to 2 equals 4; 3 to 4 equals 4; 5 to 6 equals 1; 7 to 8 equals 1; 9 to 10 equals 1; 16 to 20 equals 1; 26 to 30 equals 1; 31 to 35 equals 1; 51 to 100 equals 5; 101 to 150 equals 2. Highest bar value is 9 at 0, followed by 5 at 51 to 100. 2012. The y-axis label is No. of mice. The y-axis range is 0 to 14. The x-axis label is Range of worm burden. The x-axis categories are: 0, 1 to 2, 3 to 4, 5 to 6, 7 to 8, 9 to 10, 11 to 15, 16 to 20, 21 to 25, 26 to 30, 31 to 35, 36 to 40, 41 to 45, 46 to 50, 51 to 100, 101 to 150, 151 to 200, 201 to 250, greater than 250. Bars shown at: 0 equals 7; 1 to 2 equals 12; 3 to 4 equals 1; 11 to 15 equals 4; 16 to 20 equals 3; 21 to 25 equals 3; 26 to 30 equals 2; 31 to 35 equals 2; 36 to 40 equals 1; 41 to 45 equals 1; 51 to 100 equals 2. Highest bar value is 12 at 1 to 2. Across the four images, the largest counts occur at 0 or 1 to 2 in 2004, 2008 and 2012, while 2000 has multiple categories with a maximum value of 2.

Navigate back to Figure 2.

## Figure 3 Long description

Four bar graphs labeled A. 2000, B. 2004, C. 2008 and D. 2012. Shared x-axis label: Range of worm burden. Shared x-axis categories: less than 1; 1 to 5; 5.1 to 10; 10.1 to 15; 15.1 to 20; 20.1 to 25; 25.1 to 30; 30.1 to 35; 35.1 to 40; 40.1 to 45; 45.1 to 50; 50.1 to 100; 100.1 to 150; 150.1 to 200; 200.1 to 250; greater than 250. A. 2000: Y-axis label No. of mice, range 0 to 12. Non-zero bars: less than 1 equals 8; 1 to 5 equals 6; 5.1 to 10 equals 2; 10.1 to 15 equals 2; 30.1 to 35 equals 1. Highest bar is less than 1 at 8. B. 2004: Y-axis label No. of mice, range 0 to 12. Non-zero bars: less than 1 equals 9; 1 to 5 equals 8; 5.1 to 10 equals 6; 10.1 to 15 equals 3; 15.1 to 20 equals 2; 20.1 to 25 equals 1; 25.1 to 30 equals 1; 35.1 to 40 equals 1; 40.1 to 45 equals 1. Highest bar is less than 1 at 9. C. 2008: Y-axis label No. of mice, range 0 to 16. Non-zero bars: less than 1 equals 15; 1 to 5 equals 8; 5.1 to 10 equals 5; 10.1 to 15 equals 4; 15.1 to 20 equals 3; 20.1 to 25 equals 2; 25.1 to 30 equals 1; 35.1 to 40 equals 1; 40.1 to 45 equals 1. Highest bar is less than 1 at 15. D. 2012: Y-axis label No. of mice, range 0 to 20. Non-zero bars: less than 1 equals 13; 1 to 5 equals 8; 5.1 to 10 equals 2; 10.1 to 15 equals 1; 40.1 to 45 equals 1; 100.1 to 150 equals 1. Highest bar is less than 1 at 13. Across A. 2000, B. 2004, C. 2008 and D. 2012, the largest counts occur in the lowest worm-burden categories less than 1 and 1 to 5. Higher worm-burden categories appear as single-count bars in a small number of bins, including 30.1 to 35 in A. 2000, 35.1 to 40 and 40.1 to 45 in B. 2004, 35.1 to 40 and 40.1 to 45 in C. 2008 and 40.1 to 45 and 100.1 to 150 in D. 2012. Each year uses a different bar fill style to distinguish the four graphs.

Navigate back to Figure 3.

## Figure 4 Long description

Title text “A. Males”. A bar chart with a legend listing “Observed” and “Expected”. The horizontal axis label reads “Range of worm burden”. The vertical axis label reads “No. of mice”. The vertical axis shows 0 to 50. The horizontal axis categories read: 0, 1 to 5, 6 to 10, 11 to 15, 16 to 20, 21 to 25, 26 to 30, 31 to 35, 36 to 40, 41 to 45, 46 to 50, 51 to 100, 101 to 150, 151 to 200, 201 to 250, greater than 250. Bars are plotted for the categories, with the tallest bar at 0 and progressively smaller bars across higher ranges. A smooth curve labeled “Expected” starts near the top of the vertical scale at the left and declines toward the baseline as the worm burden range increases. Title text “B. Females”. A bar chart with a legend listing “Observed” and “Expected”. The horizontal axis label reads “Range of worm burden”. The vertical axis label reads “No. of mice”. The vertical axis shows 0 to 50. The horizontal axis categories read: 0, 1 to 5, 6 to 10, 11 to 15, 16 to 20, 21 to 25, 26 to 30, 31 to 35, 36 to 40, 41 to 45, 46 to 50, 51 to 100, 101 to 150, 151 to 200, 201 to 250, greater than 250. Bars are plotted for the categories, with the tallest bar at 1 to 5 and additional bars across multiple higher ranges. A smooth curve labeled “Expected” starts higher on the left and declines toward the baseline as the worm burden range increases.

Navigate back to Figure 4.

## Figure 6 Long description

A showing a stacked bar graph with a legend listing Wadi Tlah and Wadi Gharaba. The vertical axis is labeled No. of mice, ranging from 0 to 18. The horizontal axis is labeled Biomass (mg) class range, with categories from 1 to 5.0 through greater than 1000.0. The tallest stacked bar is in the greater than 1000.0 category, reaching about 17 mice total. Several midrange categories between 50.1 to 100.0 and 300.1 to 350.0 show stacked bars reaching into the low to mid teens. Many categories from 1 to 5.0 through 50.0 show stacked bars mostly below 10 mice. Both Wadi Tlah and Wadi Gharaba appear within multiple biomass classes, including the highest class. B showing a scatter plot with a legend listing Wadi Gharaba, Linear (Wadi Gharaba), Wadi Tlah and Linear (Wadi Tlah). The horizontal axis is labeled No. of worms recovered, ranging from 0 to 140. The vertical axis is labeled Weight (mg), ranging from 0 to 1600. Two dotted linear trend lines rise from lower left toward upper right. Points for Wadi Tlah and Wadi Gharaba are plotted, with many points clustered between 0 and 40 worms recovered and 0 to 400 mg. Higher values extend to about 120 to 130 worms recovered with weights near about 1000 to 1400 mg. The two graphs present biomass distribution by class in A and the relationship between worms recovered and weight in B.

Navigate back to Figure 6.

## Figure 7 Long description

Four scatter plots illustrate the relationship between worm burdens and biomass in male and female mice against PC1, a proxy for host age. In plot A, the x-axis represents PC1 and the y-axis represents the number of worms in male mice. The data shows a clustering of points near zero with some higher values as PC1 increases, indicating a positive correlation between worm burden and host age. Plot B shows the x-axis as PC1 and the y-axis as worm biomass in milligrams for male mice. There is a similar pattern of clustering near zero, with a few higher biomass values at higher PC1, suggesting increased biomass with age. Plot C presents the x-axis as PC1 and the y-axis as the number of worms in female mice. The distribution is similar to plot A, with clustering near zero and some higher values at increased PC1, indicating a positive correlation. Plot D shows the x-axis as PC1 and the y-axis as worm biomass in milligrams for female mice. There is a noticeable increase in biomass at higher PC1 values, with a dense cluster near zero and a sharp rise in biomass, especially in older mice. The plots highlight differences in worm burdens and biomass between male and female mice as they age.

Navigate back to Figure 7.

## Figure 8 Long description

Twelve bar graphs display the frequency distributions of worms by weight classes in milligrams for selected mice. Each graph corresponds to a specific mouse, labeled in the top left corner. The horizontal axis represents weight classes ranging from 0 to 30 milligrams in 2 milligram intervals, while the vertical axis shows the number of worms, ranging from 0 to 25. 1. Mouse 150: Peaks at 4 and 6 milligrams with a narrow distribution. 2. Mouse 208: Peaks at 2 milligrams, showing a narrow distribution. 3. Mouse 156: Peaks at 6 milligrams, with a broader spread. 4. Mouse 158: Peaks at 8 milligrams, with multiple smaller peaks. 5. Mouse 155: Peaks at 10 milligrams, showing a wide distribution. 6. Mouse 230: Peaks at 4 milligrams, with a narrow spread. 7. Mouse 161: Peaks at 6 milligrams, with a moderate spread. 8. Mouse 115: Peaks at 8 milligrams, with multiple peaks. 9. Mouse 229: Peaks at 2 milligrams, showing a narrow distribution. 10. Mouse 121: Peaks at 6 milligrams, with a moderate spread. 11. Mouse 155: Peaks at 10 milligrams, showing a wide distribution. 12. Mouse 183: Peaks at 8 milligrams, with multiple peaks. The graphs highlight differences in worm weight distributions among the mice, with some graphs showing concentrated peaks in specific weight classes and others displaying more spread-out distributions. No visual differentiation in bar style is noted across the graphs.

Navigate back to Figure 8.

## Table 3 Long description

Measures of parasite aggregation are reported by site, year, age class, sex, and for all data combined, using three aggregation metrics plus the count and percent of mice carrying about 60 percent of worms. By site, Wadi Gharaba shows higher aggregation than Wadi Tlah, with the variance to mean ratio 53.65 versus 32.53 and a slightly lower dispersion index, 0.647 versus 0.719; the negative binomial exponent is 0.417 versus 0.321. Across years, Year 2004 has the highest variance to mean ratio at 69.78, while Year 2000 is lower at 39.31 and Year 2008 is 44.08; dispersion index values are similar across years, roughly 0.668 to 0.721. By age, Class 2 is more aggregated than Class 1, with variance to mean ratio 52.27 versus 26.77 and a higher negative binomial exponent, 0.526 versus 0.158. By sex, females show much higher variance to mean ratio than males, 63.45 versus 19.09, while dispersion index values are close, 0.711 versus 0.679. The proportion of mice carrying about 60 percent of worms varies modestly, from 7.0 percent in Age Class 1 up to 17.9 percent in Wadi Gharaba; combined data report 31 mice, 12.9 percent, carrying 60.2 percent of worms. Comparisons should be interpreted with awareness that sample sizes differ by subgroup and that the aggregation metrics summarize distribution shape rather than total worm burden.

Navigate back to Table 3.
